# The redesign of the medical intern assignment mechanism in Israel

**DOI:** 10.1186/s13584-015-0014-y

**Published:** 2015-03-25

**Authors:** Alvin E Roth, Ran I Shorrer

**Affiliations:** Department of Economics, Stanford University, Stanford, CA USA; Department of Economics, Harvard University, Cambridge, MA USA

**Keywords:** Internship, Lottery, Market design, Israel, Medical graduates

## Abstract

A collaboration of medical professionals with economists and computer scientists involved in “market design” had led to the redesign of the clearinghouse assigning medical students to internships in Israel. The new mechanism presents significant efficiency gains relative to the previous one, and almost all students get a better chance of getting what they want. Continued monitoring of the new mechanism is required to verify that it is not abused, and explore whether it can be improved. Other organizations in Israel may also be able to profit from the experience that accumulates from market design, both in Israel and abroad.

The paper by Bronfman et al. [1] reports on a redesign of the clearinghouse that assigns medical students to internships in Israel. The new system, used for the first time in 2014, is the result of a collaboration of medical professionals with economists and computer scientists involved in “market design”.

Market design is an emerging subfield of economics, and market designers have helped design or redesign a variety of markets around the world. Many of these markets, like the market for internships, are *matching markets*, in which money does not play a decisive role, and may play no role at all, in determining who gets what.

Examples in the United States include entry level labor markets for physicians like the National Residency Matching Program (NRMP) [2] and a variety of medical fellowship markets [3,4]; clearinghouses for organ transplants through kidney exchange [5,6]; and clearinghouses for assigning public school positions in several American cities including New York [7]. One thing that practical market design has taught us is that a new design is often shaped to a significant extent by existing arrangements [8,9].

Bronfman et al.’s project began with the observation that the old method of assigning medical interns to hospitals in Israel didn’t work as well as it could, in the sense that it was possible to give many students better chances of getting the internships they wanted, without giving any other students worse chances. One requirement that was placed on any new system was that, any time it was used, the new assignment mechanism would in fact give all or almost all students a better chance of getting what they wanted than they would have gotten if the old system had been used instead.

The previous method of assigning interns, before 2014, was what economists call a *random serial dictatorship* (RSD). Under RSD, a lottery is conducted to randomly draw an ordering of students, and then each student chooses, in order when his or her turn arises, from those internship positions that are still available. For a number of years before the old system was computerized, the way the mechanism was implemented in the Israeli internship market was that students gathered in an auditorium where ID numbers were drawn from a hat. Clearly, after the fact, if no one’s circumstances change (thus changing their preferences) there would be no pair of medical students who would have wanted to switch their placements with each other (since the one who got to choose earlier could have simply chosen her colleague’s position when it was her turn, if she preferred it, but she did not). This property is known as *ex-post efficiency*.

But Bronfman et al. knew that ex-post efficiency—the absence of easy trades *after* positions have been assigned by RSD, isn’t the same as the absence of possible trades *before* choices are made [10]. Before the actual assignments of internships were made, each student was in fact assigned a lottery among possible positions, which would depend on that student’s choice position in the lottery, but also on which students got to choose earlier. That is, what positions would be available when a given student was asked to choose would depend not only on his or her own lottery number, but also on who had been invited to choose earlier, and what positions they had chosen and were hence no longer available. As Bronfman et al. show through an example, this makes it possible to trade *probabilities*, in a way that gives medical students better chances of getting positions they prefer.

Loosely speaking, the new mechanism operates by first conducting the old mechanism many times, using the preference lists that students have submitted to determine what internships they would choose on their turn, to discover what probability each student has of getting each internship. Then the mechanism executes trades—of probabilities—that give students a better chance of getting a more preferred internship, under the constraint that no student gets worse probabilities than they would have if the old system had been used. So, compared to the old mechanism, more students will get their first choice, more will get their first or second choice, and so on (which is called *rank efficiency*, since the distribution of rank orderings is improved [11]). Sophisticated mathematical tools are needed to accomplish this, including a celebrated result called the Birchoff-von Neumann theorem [12-14]. Bronfman et al. show that their approach markedly outperforms the RSD benchmark.

Centralized clearinghouses to which participants report their preferences have an additional benefit; they allow the community to learn about the desirability of different options according to submitted preferences. Policy-makers and future users of the mechanism may find this information valuable. A second contribution of the current paper is in proposing a novel approach for ranking the desirability of hospitals from the interns’ point of view. The proposed approach improves upon the existing one in several dimensions.

The importance of this paper stems mostly from the fact that internship placement has a significant effect on medical graduates. This is also the reason that many medical students seek advice on how to deal with the allocation mechanism. In this regard, the new mechanism lacks one of the desirable properties of the previous one – making it completely safe to report one’s true preferences.

A basic observation about RSD is that medical students can never receive a less desired internship by submitting a rank order list that doesn’t correspond to their actual preferences. (The only role of submitted preferences in RSD comes at the moment when a student chooses an internship from the available set and the student gets what he or she chooses.) This property is not completely satisfied by the new mechanism, since students receive probabilities that can be traded. It is not at all clear that the theoretical possibility to manipulate the mechanism would be realized in practice, and constitute an actual problem [15]. But it will be desirable to continue to monitor how the mechanism performs—the need for continued monitoring is another quite general lesson of market design, incidentally.

The redesign of the Israeli medical internship market can be viewed within the larger context of the design of entry level labor markets and other matching markets. The Israeli internship market is special in several dimensions. Unlike with internships and residencies in the US, hospitals have no say in the process. This is what allows probabilities to be traded among students, based only on students’ preferences over hospitals, since no information about hospitals’ preferences over students is considered.

There are other markets in Israel that could benefit from re-design, and in some of those markets both sides of the market have preferences over participants on the other side. One example we are aware of (and on which we understand some market design is already underway) is the match of students and clinical psychology programs. In this market, not only do students have preferences over programs, but each program has its own preferences over students. Settings like this one have been treated extensively by market designers around the world, and the accumulated knowledge offers some guidelines for the design of such systems.

Some thought is also presently being given in Israel to the way school slots are assigned to children, and the way organs for transplantation are allocated. Other markets, like the internship market for lawyers, suffer from problems that have been observed and sometimes dealt with in similar markets in other countries. So other organizations in Israel may also be able to profit from the experience that accumulates from market design, both in Israel and abroad.
